# Curriculum Innovation: The Impact and Benefits of Cross-Disciplinary Educational Collaboration Between a Department of Neurology and Department of Drama

**DOI:** 10.1212/NE9.0000000000200361

**Published:** 2026-09-18

**Authors:** Erica Nerbonne, Scott Magelssen, Wolfgang G. Muhlhofer

**Affiliations:** 1 School of Drama, University of Washington, Seattle; and; 2 Department of Neurology, University of Washington, Seattle.

## Abstract

**Introduction and Problem Statement:**

Simulation-based education is increasingly used in neurology training. Incorporating drama students as simulated patients and family members is a feasible approach to high-fidelity simulation, but formal evaluations and longitudinal outcomes of cross-disciplinary educational collaborations are limited. To address this gap, our neurology and drama departments jointly developed a simulation curriculum and assessment of its impact on educational outcomes of residents, and healthcare perception and performance skills of drama students.

**Objectives:**

Objectives of the curriculum were to provide hands-on practice with direct feedback, build confidence in skills and knowledge, foster long-term changes in behaviors and attitudes, evaluate cross-disciplinary simulation as an instructional method, and establish collaborative simulation-based education.

**Methods and Curriculum Description:**

A needs assessment of residents, alumni, faculty, and stakeholders informed curriculum development for neurology residency and undergraduate drama programs. Faculty–senior resident dyads developed inpatient and outpatient simulation scenarios. Drama students received presimulation coaching and portrayed patients and family members. Both groups participated in joint debriefings. We assessed educational outcomes using immediate and follow-up surveys, analyzing quantitative data descriptively and qualitative responses via directed content and thematic analysis using the Kirkpatrick framework.

**Results and Assessment Data:**

From June 2023 to December 2025, 41 neurology residents participated in 40 simulations as observers or active participants. The simulations incorporated 32 drama students. A total of 258 postsession evaluations were collected from residents and faculty and demonstrated high curriculum effectiveness with 83% to 91% of neurology residents and 98% to 100% of faculty recommending outpatient and inpatient simulations, respectively. Follow-up surveys were submitted by 61% of neurology residents and 62% of drama students. More than two-thirds (80%) of residents reported encountering at least 1 clinical case resembling a simulation within the subsequent 3 to 12 months, providing greater confidence in counseling patients and interpreting diagnostics. The vast majority (93%) of drama students recommended participation, 68% reported using skills developed during simulation in subsequent performances, and 85% reported a positive shift in perspective of healthcare professionals.

**Discussion and Lessons Learned:**

This simulation curriculum was associated with high learner satisfaction and perceived long-term transfer of clinical, communication, and performance skills among neurology residents and drama students. Our findings suggest cross-disciplinary simulation supports learning for both groups and provides a replicable model for collaborative neurology education.

## Introduction and Problem Statement

Efficient neurologists are well trained in the care of neurologic diseases encountered in both inpatient and outpatient settings^1,2^ and have perfected their focused neurologic history and examination skills.^3,4^ In an ideal world, all of these aspects of neurologic care should be practiced and refined under guidance of experienced clinicians who observe trainees and provide immediate and actionable feedback.^5-7^ Unfortunately, most neurology residency programs tend to be inpatient and acute care focused^8-10^ and higher patient volume and complexity, paired with economic pressure, rarely leave enough time to do exactly that. Instead, trainees tend to get feedback in a delayed or generic fashion, if they get any constructive feedback at all.^11-14^

Simulation-based education (SBE) as a curricular component of neurology residency lends itself as a potential solution to all these challenges. There is a growing body of literature supporting SBE as an effective and innovative method of instruction in neurology.^15^ SBE has its foundation in the experiential learning cycle, which assumes that “knowledge is created through the transformation of experience.”^16^ Well-facilitated debriefings based on direct observation of a trainee's performance can address knowledge gaps, uncover misconceptions, and reshape a trainee's attitudes and behaviors.^17,18^ Furthermore, SBE trains learners about the important concepts of teamwork, interpersonal engagement, and communication in a psychologically safe space.^16,19,20^ At the same time, the literature on long-term impact of SBE on neurology trainees' behavior and attitudes in real-life clinical scenarios remains limited,^15,18,19^ and SBE (especially high-fidelity simulation including the utilization of standardized patients) can be resource and time intensive.^15,21^

Collaborations between health care programs and drama departments have increasingly been explored as a method to enhance SBE, serving as a more cost-effective alternative to professional standardized patients.^22-25^ Universities have used drama students in health care simulations for undergraduate and graduate medical education.^22,26-28^ In these programs, trainees interact with drama students performing as patients, family members, or caregivers in scripted or improvised clinical scenarios, followed by structured debriefs to reflect on communication and decision-making skills.^22,28,29^ Drama students contribute to realistic scenarios, promote learner engagement, and foster communication and empathy among medical providers.^22,28,30^ At the same time, their participation raises questions about actor training, affective labor, and the educational value of these experiences for drama students.^22,30^ Addressing these concerns requires a “mutual pedagogy” in which health care and theater practitioners learn from each other's epistemologies and simulation training benefits both groups.^31^ The educational impact of cross-disciplinary simulation models for both neurology residents and drama students has not been systematically evaluated in peer-reviewed literature.^27,28^

## Objectives

We are presenting a longitudinal, simulation-based curriculum with high-fidelity simulation scenarios set in both inpatient and outpatient care settings. It combines pertinent approaches to diagnosis and treatment of chronic neurologic diseases and emergencies across the broad range of neurologic subspecialties with important concepts of teamwork, interpersonal, and communication skills. Furthermore, under the guidance of drama faculty, the curriculum includes dedicated coursework to coach acting students on signs and symptoms of neurologic disease and character development representing an essential part of the high-fidelity simulations.

The curriculum has the following overarching objectives for both neurology and drama trainees:

1. To provide hands-on practice and direct, timely feedback on their performance.
2. To build confidence in their existing skills and knowledge.
3. To foster meaningful and long-term changes in real-life behaviors and attitudes.
4. To evaluate simulation as an instructional method across educational disciplines.
5. To establish a culture of simulation-based education that connects departments at our institution and contributes to increased trust and empathy for health care workers.

We present 3 years’ worth of evaluation data providing Kirkpatrick levels 1, 2, and 3 evidence of long-lasting effectiveness of this cross-disciplinary curriculum for both parties involved.

## Methods and Curriculum Description

### Needs Assessment and Topic Selection

In January 2023, we conducted an online survey inquiring about current feedback culture, preferred methods of instruction, and most relevant clinical and communication skills that would benefit from direct observation and feedback in a simulated environment. The survey was distributed to 45 active neurology residents and interns, 37 alumni residents, and 115 active neurology faculty.

Responses were received from current residents and interns (42%), alumni residents (32%), and faculty (15%). There was an almost even split between junior (PGY-1/2) and senior residents (PGY-3/4). Among clinical faculty, almost 60% had more than 16 years of cumulative clinical experience, and 82% spent most of their time in clinical care including inpatient and outpatient settings. Among these responses, 26%–35% of residents and 47%–53% of faculty reported that there was insufficient feedback on clinical and communication skills. All respondents agreed that hands-on experience in a clinical or simulated setting along with direct observation and feedback from faculty and senior residents were the most effective methods of instruction. Survey results were discussed with educational stakeholders in the adult neurology residency program and compared with published literature.^10-14^ Based on these results, clinical, communication, and interpersonal skills were identified and paired with high-yield disease entities covering the entire range of neurologic subspecialties. High-yield disease entities were determined based on topics suggested in free-text comments of the needs assessment and in discussion with residency program leadership and core faculty, who represent core members of the clinical competency committee, and senior residents in charge of development and delivery of the longitudinal residency curriculum.

### Scenario Development

Once identified, each topic was assigned to a total of 22 faculty and chief resident dyads. Faculty were selected based on their clinical expertise and teaching experience in their subspecialty field. Each dyad was first tasked to define 3 to 4 specific, measurable, achievable, relevant, and timely learning objectives for their assignments.^32^ At least 1 objective was required to pertain to communication skills. Dyads were then provided a standard simulation flowsheet to fill out (please see eAppendix 1 for details of the scenario flowsheet template) and tasked to provide a history and physical exam script for drama students acting as simulated patients, family members, or caregivers. Furthermore, where appropriate, a detailed description of the patient's or family member's health and psychosocial background was provided. Finally, dyad teams were tasked to create a slide deck with pertinent clinical and diagnostic data. Based on the learning objectives and templates above, one of the authors (W.G.M.) created an observational checklist and debriefing templates for each case to support content experts tracking critical learners' activities and simulation facilitators conducting a structured debrief.

A list of the final simulation scenarios and respective learning objectives is presented in eTables 1 and 2. Difficulty levels of simulations were adjusted for trainees' experience, either by making neurologic presentations less obvious, adding medical complications, or incorporating psychosocial or communication challenges to respective cases. Details and materials for each individual case can be provided by the authors upon direct request.

### Simulation Setup and Curriculum Structure

Instead of mannikins, most simulations included simulated patients, and in some cases family members or caregivers, to maximize fidelity and trainee engagement and to provide hands-on practice and feedback on neurologic examination and communication skills. All simulations were conducted at our institutional simulation center with most rooms being equipped with screens to project imaging and laboratory data, audio-visual recording capabilities, and remote vital signs control.

The curriculum sorted the entire resident cohort into junior (PGY2) and senior residents (PGY3 and 4). In the study time frame from June 2023 to December 2025, the annual junior resident cohort consisted of 8–10 and the senior resident cohort of 16 trainees per year. On the day of simulations, each neurology resident cohort was divided into 2 fairly equal-sized groups (4–8 residents per group). Two cases were always held simultaneously in separate rooms. One to 2 trainees were actively engaged in the simulation and the remainder observed. Trainees were asked to take turns being participants and observers so that on average, each trainee had a chance to actively participate in at least 1 to 2 cases over the course of the curriculum. Each cohort had 1 hour per case: 15–20 minutes of simulation and 40–45 minutes of debriefing. Each simulation had either a neurology chief resident or teaching faculty acting as an embedded simulation person (ESP). The ESP was familiar with the simulated scenario, disclosing test results, providing cognitive aids, and administering treatments on trainees' request. The ESP also functioned as debriefing facilitator and underwent dedicated training on the modified Promoting Excellence And Reflective Learning in Simulation and “debriefing with good judgement” debriefing framework, provided by the author W.G.M., who holds a formal Master's degree in Healthcare Simulation and has performed extensive faculty development on various debriefing techniques.^33,34^ Content experts were asked to observe the simulation either in person or remotely through live video and audio streaming and then joined for the in-person debriefing discussion. Both content experts and ESPs were asked to use the case-specific observational checklists and debriefing guides generated by the author W.G.M. to keep the teaching experience standardized across groups and cases. Please see eAppendices 2 and 3 for details of the standardized debriefing template and guide and the observational checklist template and guide. Finally, a simulation operating technician adjusted vital signs displayed on monitors in real-time as needed.

### Drama Student Selection, Coaching, and Course Assignments

Students enrolled in the drama major or minor at our institution were recruited on a volunteer basis through email. One of the authors (W.G.M., a board-certified neurologist with over 15 years of experience in clinical neurology care) met with each student about a week ahead of their sessions for 1-hour, one-on-one coaching sessions on their neurologic history and examination, background story, and subtext. Drama students portraying family members and caregivers received their own script with background story and subtext including their emotions, worries, and challenges and were either coached separately or in conjunction with patient actors. Each student had the option to claim credits towards their acting program by submitting a self-reflective paper after their participation. For the reflection, students were asked to provide a detailed account of their coaching experience, their character building, and interaction with neurology trainees during the simulation. Furthermore, they were tasked to describe whether and how they drew on previous drama experience and how this experience compared with other kinds of drama performances. Finally, the drama students had to describe potential benefits of collaborative training between drama students and medical professionals. One of the authors (S.M., faculty in the drama department) provided direct feedback on the self-reflection papers.

### Collection of Immediate Postsession and Follow-Up Evaluation and Feedback

Evaluations were collected from faculty, neurology residents, and drama students immediately after the simulations using QR-based online surveys.

For neurology residents, faculty, and simulation facilitators, the immediate postsession surveys consisted of 5-point Likert scale items, categorical questions, and open-ended prompts assessing achievement of learning objectives, communication skills, realism and engagement, debriefing quality, and overall educational value. Additional items evaluated the perceived appropriateness of the simulation duration and difficulty level. For drama students, additional items evaluated the usefulness of presimulation coaching, ability to portray and communicate their characters effectively, application of theatre and improvisation skills, the contribution of character traits to scenario authenticity, perceived growth as actors, understanding of communication challenges in clinical settings, and quality of interactions with residents. Open-ended questions invited students to comment on benefits of simulated patient work, applicability to other professions, and whether they would recommend the experience.

At 3 years, a follow-up survey was disseminated electronically through email and text message and assessed potential long-term impact on neurology trainees and drama students.

The survey asked neurology residents whether they had subsequently encountered real-world clinical or professional situations reminiscent of the simulated scenarios. Specifically: which elements of those encounters overlapped with the simulations (e.g., diagnostic challenges, communication with patients or families, and interprofessional interactions), and which teaching points or skills learned during the simulations were applied in real clinical practice. Respondents were also asked to describe memorable simulation scenarios and the lessons that remained salient years later.

On the drama student side, items asked whether respondents had encountered situations resembling the simulated scenarios and which skills they continued to apply—such as script analysis, improvisation, emotional awareness, embodiment of symptoms, collaboration with professionals, and integration of feedback. Additional questions explored memorable scenarios, enduring takeaways, and any shifts in perceptions of health care, including increased empathy, awareness of communication and diagnostic complexity, and insight into interprofessional dynamics. Respondents also identified specific aspects of the simulation—such as portraying symptoms, observing communication styles, and receiving clinician feedback—that shaped these perceptions.

### Data Analysis

Quantitative data were summarized descriptively.

Survey responses from residents and faculty underwent 2 independent reviews by authors E.N. and W.G.M., who merged their narrative summaries and discussed potential discrepancies. Free-text data from residents and faculty were categorized using direct content analysis guided by the Kirkpatrick framework.^35^ Because respondents could contribute free-text responses across multiple survey items, frequency counts were based on unique respondents providing at least one narrative response.

Drama student free-text responses from surveys and self-reflection essays were analyzed using reflexive thematic analysis with inductive coding.^36^ Free-text responses were analyzed thematically and used alongside reflective essays to contextualize and elaborate initial themes due to content overlap and limited depth. Data were reviewed at the level of individual responses, with inductive codes generated from meaningful text segments and iteratively refined into broader themes. Coding and preliminary theme development were conducted by E.N. Final themes were discussed among all authors and mapped to Kirkpatrick levels of educational outcome.

### Standard Protocol Approvals, Registrations, and Participant Consents

All procedures complied with institutional review board guidelines, which deemed the study exempt based on the fact that our research was conducted in commonly accepted educational settings and involved normal education practices that were not likely to adversely affect trainees' opportunity to learn required educational content or the assessment of educators who provided instruction. See eAppendices 4 to 8 for details of each survey.

### Data Availability

Anonymized data not published within this manuscript will be made available by request from any qualified investigator.

## Results and Assessment Data

From June 2023 to December 2025, 41 neurology residents were exposed to a total of 40 simulations, either as observers or active participants. The simulations incorporated 32 drama students and were facilitated by 22 individual faculty and 11 senior residents. Postsession surveys were collected from residents, who were active participants and observers and we invited both content experts and simulation facilitators (i.e., ESPs) to submit feedback through faculty surveys. This resulted in 204 individual postsession surveys from residents and 54 from faculty. Thirty of 32 drama students submitted a postsession survey. Twenty-five of 41 (61%) of neurology residents, and 20 of 32 (62%) drama students completed a follow-up survey. In addition, 27 of 32 (84%) drama students submitted written reflections on their acting experiences.

## Neurology Resident Outcomes

### Initial Reactions and Satisfaction With Curriculum (Level 1)

#### Quantitative Data on Curriculum Duration and Difficulty From Neurology Residents and Faculty

Most faculty and trainees found the difficulty of simulations appropriate, especially for outpatient scenarios. Less than 17% of trainees tended to perceive the duration of simulations and debriefings as “too long,” whereas up to 20% of faculty felt they were “too short” (Table 1).

**Table 1:** Neurology Faculty and Trainee Evaluation of Simulation Duration and Difficulty

			Duration of entire experience				
		Faculty				Trainees	
	Too long	Too short	Just right		Too long	Too short	Just right
Inpatient (n = 49)	2%	11%	87%	Inpatient (n = 180)	12.3%	2.5%	85.2%
Outpatient (n = 5)	—	20%	80%	Outpatient (n = 24)	16.7%	4.1%	79.2%

			Duration of debriefing				
		Faculty				Trainees	
	Too long	Too short	Just right		Too long	Too short	Just right
Inpatient (n = 49)	5.5%	9%	85.5%	Inpatient (n = 180)	9.9%	3.7%	86.4%
Outpatient (n = 5)	—	20%	80%	Outpatient (n = 24)	4.2%	—	95.8%

			Case difficulty				
		Faculty				Trainees	
	Too easy	Too hard	Just right		Too easy	Too hard	Just right
Inpatient (n = 49)	3.6%	7.3%	89.1%	Inpatient (n = 180)	2.5%	5.6%	91.9%
Outpatient (n = 5)	—	—	100%	Outpatient (n = 24)	4.2%	—	95.8%

#### Quantitative Data on Curriculum Effectiveness From Neurology Residents and Faculty

**Figure 1:**
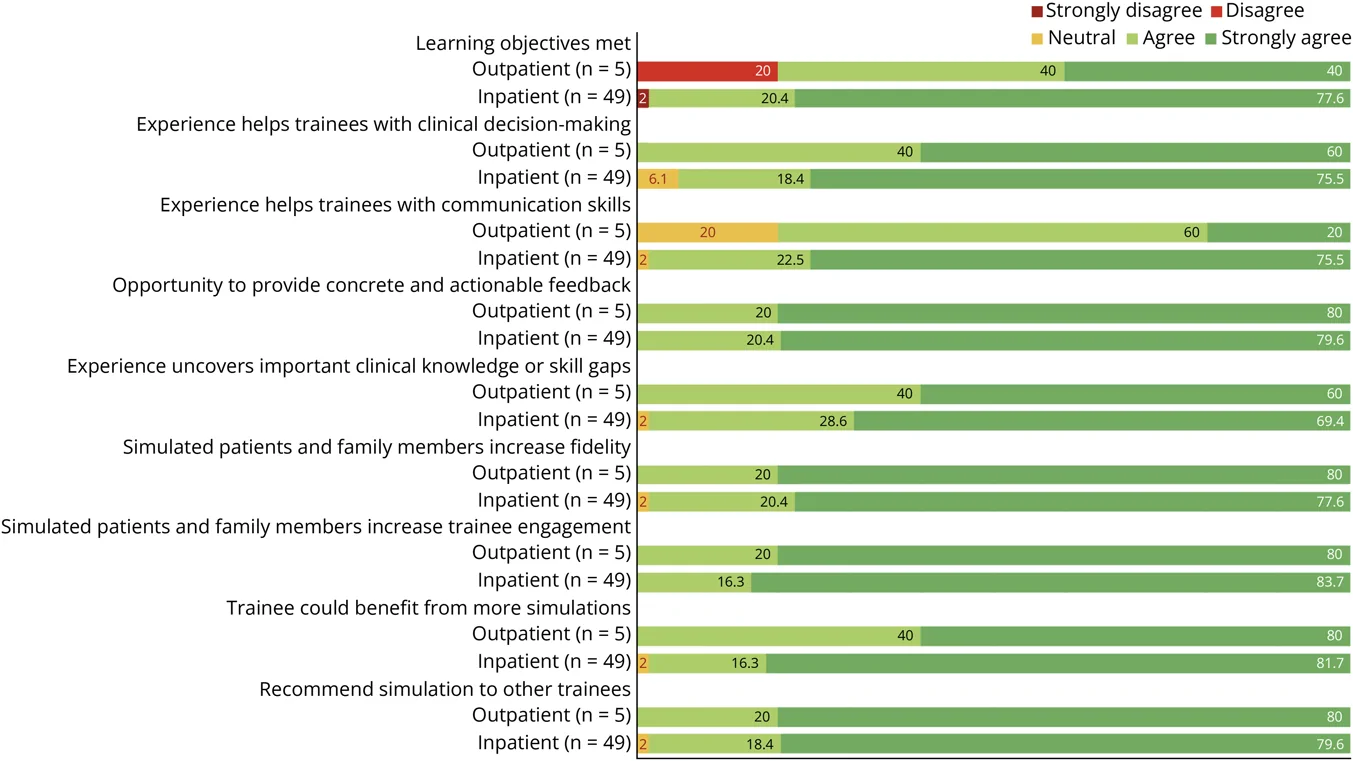
Faculty Evaluation of Teaching Method Effectiveness.

Neurology faculty and simulation facilitators (total of 54 individual responses) as well as neurology residents (total of 204 individual responses) rated simulations as highly effective, faculty giving stronger endorsements than trainees across all survey questions. Among faculty, most agreed or strongly agreed that learning objectives were met and that the simulation improved trainees' clinical decision-making and communication skills. They also valued opportunities for actionable feedback and viewed simulated patients and family members as a major strength contributing to scenario fidelity and trainee engagement. Nearly all faculty (98%–100%) would recommend the simulations for other trainees (Figure 1).

**Figure 2:**
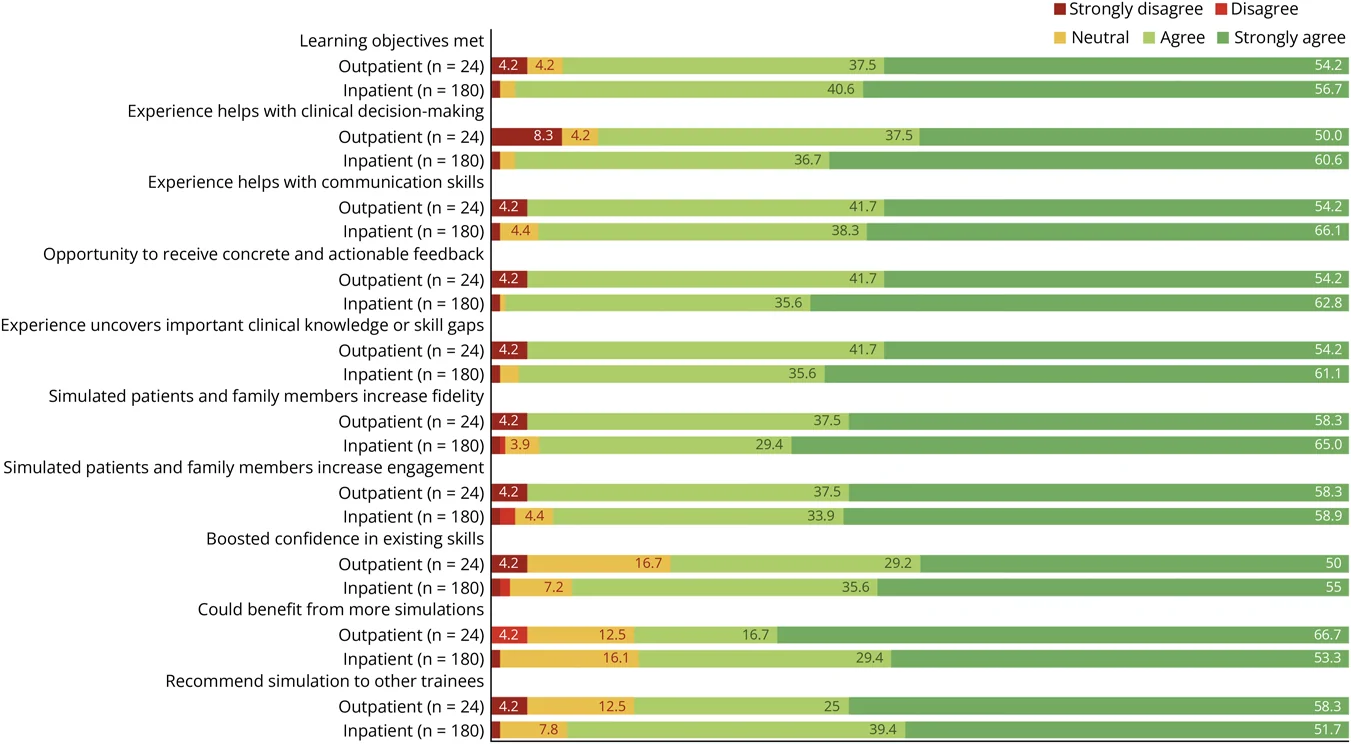
Resident Evaluation of Teaching Method Effectiveness.

Similarly, neurology residents reported favorable experiences, although with not quite as much enthusiasm and an overall trend towards favoring in- over outpatient scenarios. Yet, a strong majority (outpatient vs inpatient scenarios: 91.7% vs 97.3%) agreed or strongly agreed that learning objectives were met and that simulations refined their clinical decision-making skills (outpatient vs inpatient scenarios: 87.5 vs 97.3%). Trainees also highly valued simulated patients and family members, strongly agreeing that they increased scenario fidelity and enhanced engagement. Overall, trainees would recommend 83% of outpatient and 91% of inpatient simulations to others (Figure 2).

#### Written Feedback From Neurology Residents and Faculty

Simulation facilitators and content experts perceived simulations as more engaging than traditional didactics and valued the clinical relevance of in- and outpatient scenarios. They highlighted the authenticity of drama students' performances in supporting diagnostic reasoning and communication training. Owing to these perceived benefits, faculty recommended incorporating SBE as a core component of the neurology residency curriculum. Faculty also suggested clarifying learning objectives, better aligning case complexity with trainee experience, and enhancing scenario design, including additional debriefing time for outpatient scenarios and improved consistency in actor's performance across scenario runs.

Neurology residents valued the realistic and immersive nature of simulations. Simulations exposed residents to complex topics and provided opportunities to practice clinical and communication skills in a “safe learning environment.” Residents recommended scheduling simulations alongside relevant lectures, reducing group size, providing take-home reference materials, and allowing additional time to review outpatient workup strategies.

### Knowledge Acquisition and Skill Development (Level 2)

Most residents reported that simulations supported counseling techniques (64%). Fewer residents reported that the simulation supported interpreting diagnostic test results (48%) and ordering appropriate diagnostic tests (40%). Residents also described simulations as reinforcing diagnostic reasoning and clinical decision-making for complex cases, as well as improving communication skills and preparedness to deliver difficult news. One resident stated, “Doing the simulation with the team and having a detailed discussion about verbiage made me feel more confident in knowing what to say… I remember thinking, ‘Thank God we had that SIM lab.’”

According to respondents, high-stake scenarios and debriefing helped residents stay calm under pressure, consolidate learning, and improve team coordination. One resident reflected, “Interaction with bedside staff for collateral history and planning helped me coordinate better in real clinical settings.” Overall, trainees reported improved confidence and preparedness to approach complex neurologic encounters after the simulation.

### Application of Learning and Behavioral Change (Level 3)

Neurology residents described applying simulation lessons in real-world clinical encounters. According to residents' responses to follow-up survey questions regarding simulation-related clinical encounters and the application of simulation-derived learning in clinical practice (see Methods and eAppendices 4 to 8 for details), 80% reported having at least 1 clinical encounter in the past 3 to 12 months that reminded them of a simulation scenario. Residents also noted that many cases presented with a similar diagnostic criterion (64%), involved similar management (56%), and required similar communication strategies (52%) as simulated scenarios.

In the free-text response to the follow-up survey, a “Nearly all the simulated cases I worked on have been represented by real clinical encounters this year.” Another resident reflected, “Early in training, running a stroke code that turned out to be a seizure ended up being helpful in real cases going forward.” From these free-text responses, we identified 3 main categories that residents mentioned: clinical prioritization and decision making, communication in complex clinical encounters, and reflection and transfer of learning (Table 2).

**Table 2:** Main Categories From Residents' Follow-Up Survey Free-Text Responses (n = 20)

Category	Description	Frequency	Illustrative quotes
Clinical prioritization and decision making	Residents described simulation training as supporting the development of clinical prioritization, how to sequence clinical tasks, and real-time decision-making under pressure	70% (14/20)	“Keeping HOB elevated and neck midline as an easy first step after finding out hemorrhage is present.” “Forces you to order/do things in order rather than all at once like in real life. Helps you learn to prioritize certain things.”
Communication in complex clinical encounters	Residents highlighted simulations as valuable opportunities to practice communication in emotionally and cognitively challenging clinical situations. This included delivering bad news.	55% (11/20)	“A simulation of pseudo progression…was a great opportunity to practice delivering potentially bad, but still uncertain news to a patient.” “It was helpful to know how to approach…how to answer common questions that are always inevitably asked by a patient's family.” “[For] FND functional gait…the teaching behind breaking the news was helpful.”
Reflection and transfer of learning	Residents described simulation as a meaningful space for reflective learning, particularly through debriefing and postsimulation discussion, and reported clear transfer of learning into clinical practice	50% (10/20)	“I think the most important part of the simulation is the discussion after the fact. Thinking out loud about how to approach something is more helpful for me.” “This was over a year ago, but early in training, running a stroke code that turned out to be a seizure ended up being helpful in real cases going forward.” “Simulating difficult (or even intense) interactions with upset family members made this real-life situation somewhat easier given I had practiced it.”

“n” refers to the number of respondents who provided at least one free-text response across any survey items.

## Drama Students Outcomes

### Initial Reactions and Satisfaction With Curriculum (Level 1)

#### Quantitative Data on Curriculum Effectiveness From Drama Students

**Figure 3:**
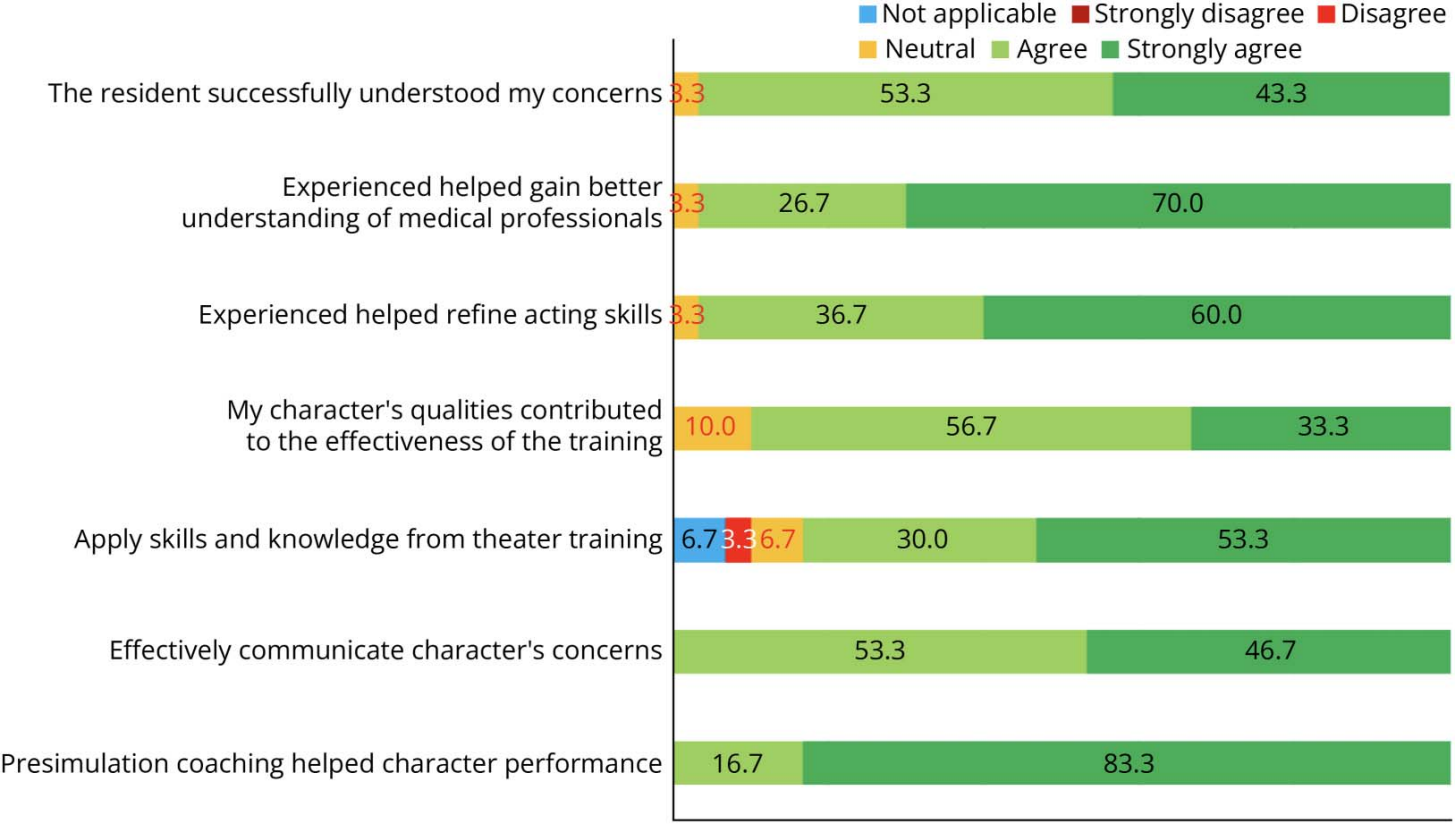
Drama Students Evaluation of Teaching Method Effectiveness All numbers above represent the percentage of total responses (n = 30).

A total of 30 of 32 (93%) drama students completed an initial survey after their simulation participation. All respondents agreed that the 1-hour coaching sessions helped them perform their characters effectively, but a few wished that they had prepared more or asked more questions in the coaching session. Over 80% agreed that the simulation provided an opportunity to apply skills from theater training. Many noted a strong sense of their value within the simulation, with their character's personality, idiosyncrasies, and emotions contributing to the training's effectiveness. Drama students also gained experience interacting with neurology residents. Most respondents (73.3%) rated their interactions with neurology residents as positive, describing them as “professional” and “caring.” When asked about broader educational and professional values, 53% indicated that all providers would benefit from simulations, with several students expressing that “all medical professionals who deal with patients” should participate. Most (93%) drama students would recommend the simulation curriculum to their peers, highlighting the benefits such as “improvising in a new context.” Overall, drama students recommended an ongoing collaboration between the neurology and drama departments (Figure 3).

### Knowledge Acquisition and Skill Development (Level 2)

Many drama students emphasized the importance of research and rehearsal strategies in creating patient portrayals. Students also reported improved ability to respond to unexpected scenarios, manage dialogue under pressure, and adjust physical and vocal behavior.

Participants also reflected on the value of experiential learning. Drama students emphasized their preparation strategies and adaptability required in simulations. A physician noted, “We do not just learn these lessons in medical school,” and a drama student echoed, “We do not just learn these lessons in drama school.”

### Application of Learning and Behavioral Change (Level 3)

In the follow-up survey, 68% of drama students reported applying skills from the simulation in later acting assignments or performances. Students noted using skills such as improvisation (57.9%) and emotional awareness (47.4%). One student commented, “I used what I learned in simulation when performing final scenes in class—it helped me navigate unexpected dialogue and maintain character authenticity.”

Many students described impacts on their artistic development and future career perspectives. They reported increased confidence, optimism, and a greater sense of preparedness for professional practice after simulation experiences. Students reflected on the experience to “hone [their] acting craft” and “learn more about the diverse ways theatre can benefit society.” As one participant noted, “the intersectionality of the medical and theater fields also reassures me that there will always be a way for artists to help the world in some way.”

**Figure 4:**
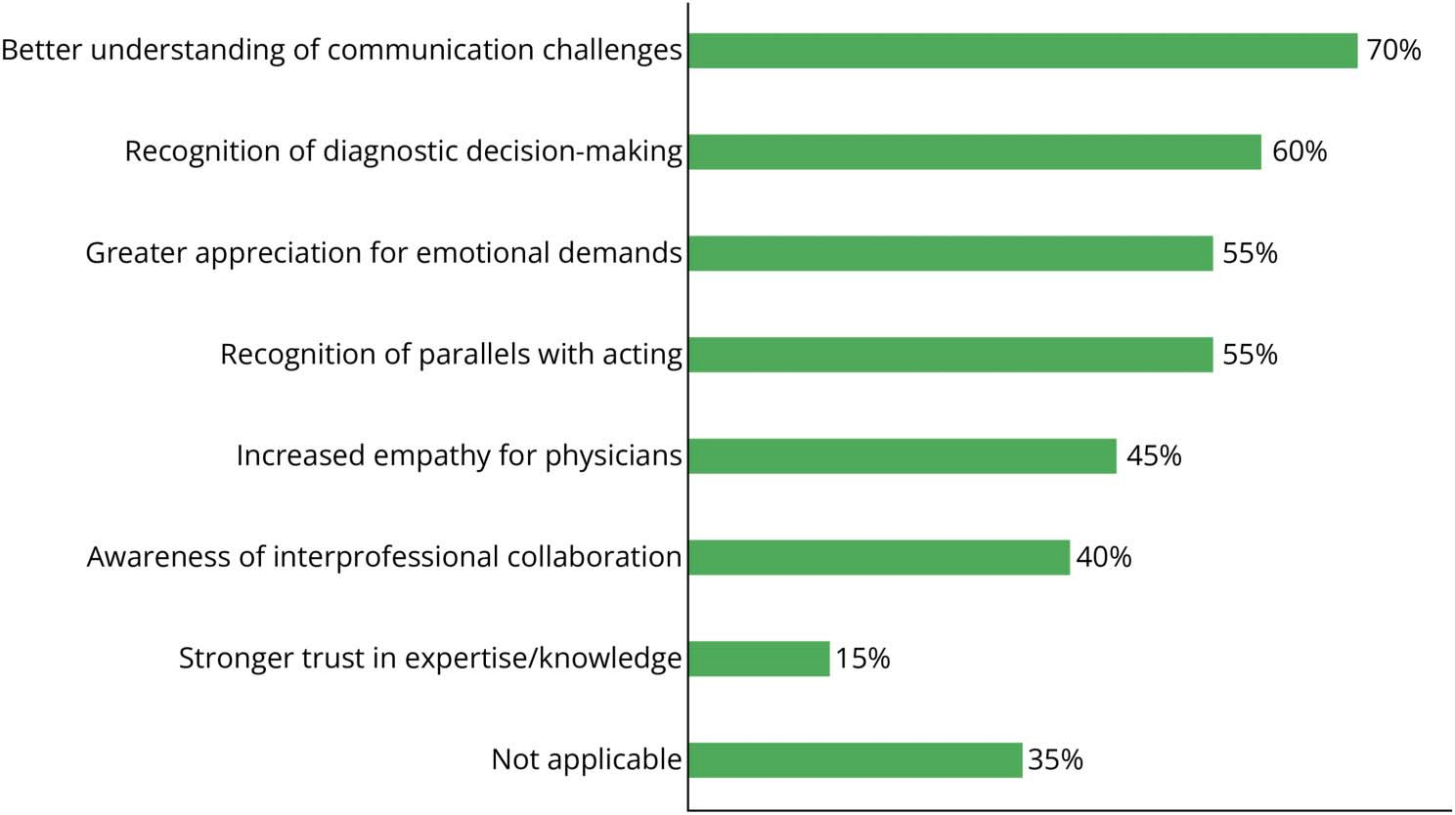
Drama Students Perspectives on Health Care All numbers above represent the percentage of total responses (n = 20).

Beyond skill development, the simulation shifted 85% of drama students' perceptions of health care professionals. By acting as patients in medical scenarios, drama students reported greater awareness of communication challenges (70%), complexities of diagnostic decision-making (60%), and emotional demands of health care (55%), as shown in Figure 4. Participants described recognizing residents as individuals navigating institutional challenges and expressed appreciation for their patient-centered communication practices, such as their openness to questions. As 1 student noted, “I have a lot of respect for them now.”

Drama students also expressed hope for the future of medicine. One student reflected, “being a part of a simulation like this made me feel like the doctors of tomorrow are going to be better.” Others suggested that future health care providers may be more equipped to address biases, particularly in gendered and reproductive care. Students also noted the value of cross-disciplinary collaboration between medicine and drama because it could reduce misunderstandings between physicians and the public.

Overall, from the self-reflections, 5 main themes were identified: preparation and character development, improvisation and adaptability, clinical realism and the ethics of representations, reflexive learning and transferable skills, and perceptions of health care professionals (Table 3).

**Table 3:** Themes From Drama Students' Written Reflections (n = 27)

Theme	Description	Frequency	Illustrative quotes
Preparation and character development	Participants described preparing for simulations through case review, coaching sessions, and development of clinically grounded patient characters based on provided materials	100% (27/27)	“I made a list of the given circumstances and noted questions about biographical gaps in the script.” “My application of Uta Hagen's ‘Nine Questions’ was one of the most impactful acting frameworks that I applied…This method encourages actors to understand their characters by asking a series of questions, such as ‘What are my relationships?’ or ‘What do I want?’.”
Improvisation and adaptability	Participants noted that simulations required real-time adjustment to unscripted questioning, examination techniques, and differing clinician communication styles	74% (20/27)	“From my patient sheet, there were certain phrases I spoke…but the rest of the answers to each question were crafted on the spot while still being consistent and in dialogue with my character's history sheet.” “I found improv to be a very useful skill…This simulation required so much improv…[and] quite a bit of sporadic movements, and I had to make it feel authentic.”
Clinical realism and the ethics of representation	Participants emphasized the importance of accurate symptom portrayal while avoiding stereotypes	66% (18/27)	“I found myself constantly deciding which symptoms to exaggerate for visibility and which ones to downplay to maintain the realism of the scene.” “The main thing I was told to avoid was pushing stereotypes. Especially given the simulated patient I was portraying was unhoused, we went into training with a compassionate approach towards a sensitive situation.”
Reflexive learning and transferable skills	Participants reported reflective learning related to performance choices, communication strategies, and development of acting skills. They also mention applying skills to future opportunities	100% (27/27)	“Upon reflection, however, I've discovered that I really can dive deep into certain aspects of character and effectively research in order to inform choices that I make in performance.” “This gave me an opportunity to test my knowledge and skills of acting that I received at the School of drama, like creating a character, using objects/tools other than their original meaning…”
Perceptions about health care professionals	Participants evaluated clinicians based on empathy, communication style, and confidence, which influenced how they perceived their interactions with residents and health care	92% (25/27)	“I remember feeling like they were very sympathetic to my situation and really wanted to reassure me and make sure that I was doing well mentally.” “I was very grateful that female reproductive health was a serious consideration when evaluating my state and medical history…” “Being a part of medical education scenarios filled me with a deeper respect, and reverence even, for the profound degree of difficulty and challenges…”

n refers to the number of respondents who provided at least one free-text response across any survey items.

## Discussion and Lessons Learned

Our evaluation data demonstrate that a cross-disciplinary SBE curriculum between the neurology and drama departments, developed through a comprehensive needs assessment and with input from content experts, is a feasible and cost-effective alternative to other high-fidelity SBE curricula using standardized patient programs.^36^ Participants perceived our curriculum as highly efficient in providing neurology trainees and drama students with hands-on practice and actionable feedback on professional skills. Across groups, participants reported improved confidence in existing knowledge and perceived long-term changes in their behaviors and attitudes. This collaboration also supported an educational culture that builds bridges across departments at our institution and increased empathy for health care workers.

Prior collaborations between health care and theatre programs have been described in nursing and undergraduate medical education, presenting simulations with drama students as an engaging pedagogical approach.^37-39^ Similar to these studies, medical learners reported perceived improvements in communication and empathy, and drama students have generally described their experience positively.^39^ Our findings extend this literature by examining educational outcomes for both learner groups, including drama students' perceptions of the impact of simulation participation on their acting skills, adaptability, professional development, and artistic practice.

Our curriculum also builds on prior efforts in neurology SBE and cross-disciplinary collaboration with drama students in other health care settings by incorporating outpatient scenarios addressing diagnostic and treatment challenges associated with chronic neurologic diseases. Although simulation-based neurology education has primarily focused on neurologic emergencies and inpatient care,^8,10,40^ outpatient neurology topics tend to be communication, rather than workup or management focused.^41-43^ Our curriculum addressed both clinical reasoning and communication objectives within the same encounter. Faculty and trainees responded positively to outpatient cases, rating the scenario difficulty as appropriate. Although 80%–90% of trainees agreed that outpatient scenarios met learning objectives, 20% of faculty disagreed, a higher proportion than for inpatient scenarios. This difference may reflect the limited time available for debriefing. Because outpatient encounters occur at a slower pace, trainees spend more time in the simulation. To address this, trainees suggested incorporating initial workup and follow-up encounters. Given our small sample size, individual responses may also disproportionately affect results. Larger studies could clarify these patterns.

Furthermore, we examined higher-level and longer-term outcomes, including enduring changes in attitudes and clinical behaviors, which are rarely included in studies on SBE or educational interventions for neurologic topics.^15,44^ These features contrast with prior simulations that focus primarily on acute inpatient care or short-term outcomes because relatively few studies have examined long-term outcomes clearly linked to a single educational intervention.^15,18,19^ Most trainees reported encountering clinical situations similar to simulated scenarios. We cannot exclude that the survey responses are confounded by at least some recollection bias because some of these scenarios might have occurred as long as 3 years ago at the time of the second survey. Clinicians tend to recall particular “good” or “bad” patient encounters and incorporate “lessons learned” into their everyday practice.^45,46^ This study also contributes to limited literature examining long-term educational impact for drama students, who reported transfer of simulation experiences to their artistic and professional practices.^25,26,31^

Several findings from our study align with literature on standardized patients, reporting improvements in learner confidence, communication skills, and perceived preparedness after simulations.^47^ Standardized patients typically undergo formalized case-specific training to ensure consistency across encounters. By contrast, our drama students received individualized coaching but were not standardized to the same extent. Consistent with standardized patient literature, our study demonstrated high learner satisfaction, increased confidence, and perceived transfer of communication and clinical skills, although evidence comparing simulation modalities remains mixed.^48,49^ However, we did not directly compare drama students with standardized patients, and our results should not be interpreted as evidence of equivalence.

Although we assessed perspective shifts only among drama students, many reported improved understanding of the emotional and cognitive demands of health care, suggesting the potential for simulations to foster respect across disciplines. Cross-disciplinary SBE programs challenge traditional silos between disciplines, such as medicine and drama, by enabling the cross-pollination of skills and knowledge in areas such as decision making and preparation. These mutual benefits are not yet well characterized across learner groups.^25,27,28,31^ Our results indicate that, with brief presimulation training, drama students can contribute meaningfully to SBE. Other institutions could adapt our design as a replicable model for integrating theater students into health care education.

### Study Limitations

This study has several limitations. Simulations occurred at a single institution with relatively small participant numbers, limiting generalizability. Faculty comparisons between inpatient and outpatient simulations should be interpreted cautiously given the small number of outpatient faculty responses, limiting the ability to draw definitive conclusions across settings. Data relied mainly on self-reported measures, which may reflect participant perceptions rather than skill development. Although SBE is increasingly valued in neurology education, it is not a standard approach.^18,49,50^ Some simulation curricula use checklist-based scoring,^49^ but because our scenarios were not highly algorithmic, checklist-guided feedback was applied without assigning numerical performance scores or threshold-based passing criteria. Although not highly rigorous, this study provides insights into how residents and drama students benefit from simulation-based education.

An additional limitation is the absence of a comparison group using professional standardized patients. As a result, this study cannot determine whether drama students produce equivalent educational outcomes compared with standardized patient models. The use of drama students may also introduce variability in symptom portrayal and simulation fidelity. To support consistency, drama students received individualized coaching and detailed case materials, and content experts who developed the scenarios and could identify inaccuracies or deviations from intended learning objectives observed simulations. Structured debriefing provided additional opportunities to address inconsistencies in case portrayal and reinforce learning objectives.

### Potential Future Directions

Although we debriefed with residents and drama students together, we did not include a formal debriefing on drama students' acting performances. In future simulations, expanding debriefings for actors could enhance their learning, increase fidelity, and offer additional insights into cross-disciplinary simulations. Scenarios could also be expanded. Additional virtual simulations could prepare residents for telehealth appointments and help actors with virtual auditions. Competitive scenarios, in which teams tackle the same time-sensitive task, could test residents' efficiency and actors' ability to stay true to scripted roles.

## Conclusion

Simulations provide neurology residents a space to practice patient encounters and develop skills for clinical practice. When designed collaboratively, these experiences benefit both residents and actors and may increase empathy for health care workers. Expanding SBE can enhance training and better prepare residents and actors for real-world practice, giving more learners the chance to think, “Thank god we had a sim lab.”

## Glossary

ESP: embedded simulation person
SBE: simulation-based education
